# Updates on thyroid disorders in pregnancy and the postpartum period

**DOI:** 10.1097/01.NPR.0000000000000130

**Published:** 2024-01-25

**Authors:** Kelly D. Rosenberger, Natalie Parker

**Affiliations:** **Kelly D. Rosenberger** is a clinical associate professor in the College of Nursing at the University of Illinois Chicago in Chicago, Ill.; **Natalie Parker** is a clinical instructor in the College of Nursing at the University of Illinois Chicago in Chicago, Ill.

**Keywords:** hyperthyroidism, hypothyroidism, postpartum thyroiditis, pregnancy, thyroid disease, thyroid disorders, thyroid function tests

## Abstract

NPs play a pivotal role in caring for pregnant people. This article provides an overview of gestational and postpartum thyroid disorders, including their assessment, management, and indications for referral. The goal of this article is to help providers better assess and manage thyroid disorders during pregnancy and improve patient outcomes.

**Figure:**
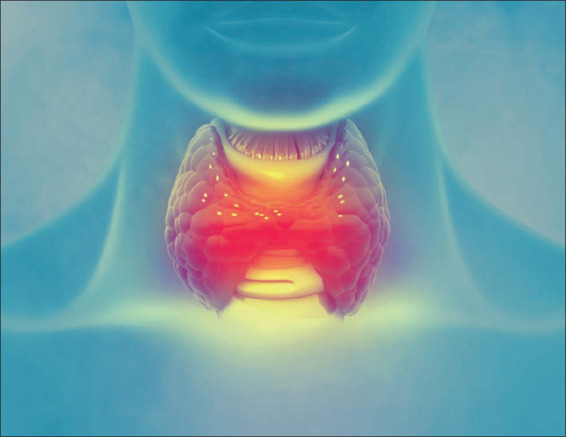
No caption available.

In females and those assigned female at birth who are of reproductive age, thyroid disorders are second only to diabetes mellitus as the most common endocrine disorders. The typical symptoms of pregnancy often emulate symptoms of thyroid dysfunction, creating a significant diagnostic challenge during this time. Poorly managed thyroid disorders during pregnancy are associated with adverse outcomes. Untreated thyroid disease during pregnancy has been associated with increased risk of miscarriage, hypertensive disorders, fetal growth restriction, placental abruption, and several other conditions that are discussed in this article.^1^,^2^ Therefore, diagnosis and treatment of thyroid disorders are necessary components of prenatal care to safeguard maternal and fetal well-being.

## Thyroid changes in pregnancy

During a typical pregnancy, increased metabolic demands significantly impact maternal thyroid physiology. The major changes in thyroid function include a slight enlargement of the thyroid gland due to hyperplasia and increased vascularity, but the enlargement does not constitute a true goiter or significant thyromegaly. Thyroid function test results change during uncomplicated pregnancies as well as in pregnancies in individuals with thyroid dysfunction; these changes vary by trimester.^3^ During typical pregnancy, concentrations of the thyroid hormones thyroxine (T4) and triiodothyronine (T3) increase; the thyroid-stimulating hormone (TSH) slightly decreases, though usually within normal range, due to human chorionic gonadotropin (hCG) cross-reacting with TSH receptors; total T4 (TT4) levels increase while free T4 (FT4) levels remain unchanged.^1^-^3^ Worldwide, the most common perinatal thyroid disorders are hypothyroidism, hyperthyroidism, postpartum thyroiditis, and goiter.^4^ In the US, where iodine intake among the general population is sufficient, goiter during pregnancy is rare. However, in iodine-deficient regions, goiter is more common during pregnancy.

## Hypothyroidism in pregnancy

Overt hypothyroidism is defined as low FT4 and increased TSH levels during pregnancy.^2^,^4^ Subclinical hypothyroidism during pregnancy is defined as increased TSH and normal FT4 levels.^1^,^2^,^4^ Worldwide, the incidence and prevalence of overt and subclinical hypothyroidism during pregnancy vary greatly due to the different definitions of each condition that have been utilized over the years in numerous studies showing inconsistent results.^2^ In the US, the highly variable recognition and treatment of subclinical hypothyroidism in pregnancy has been attributed to geographic location, socioeconomic status, race, and ethnicity.^5^ A study by Maraka and colleagues found that most pregnant patients with subclinical hypothyroidism did not receive therapy. This lack of treatment initiation was associated with both clinician and patient factors, including insufficient awareness of evolving guidelines, lack of established treatment practices, confusion due to inconsistent evidence and professional society recommendations, and patients' refusal of therapy.^5^

Globally, iodine deficiency is the most common cause of hypothyroidism generally attributed to geographic location. In countries with adequate iodine intake among the general population, the most common causes are autoimmune thyroiditis (Hashimoto thyroiditis) and iatrogenic hypothyroidism after treatment for hyperthyroidism. In the US, the American Thyroid Association (ATA) guidelines recommend supplementation with 150 mcg of iodine daily—the dose in the majority of prenatal vitamins—during pregnancy and lactation.^2^,^6^

In pregnancy, hypothyroidism has been associated with an increased risk of several complications, including preeclampsia; gestational hypertension; placental abruption; non-reassuring fetal heart rate tracings; preterm delivery, including very preterm delivery (before 32 weeks); low birth weight; increased rate of cesarean section; postpartum hemorrhage; perinatal morbidity and mortality; and neuropsychological and cognitive impairment in children.^1^-^4^,^7^,^8^

It is important for clinicians to determine if presenting signs or symptoms of thyroid disease are present. Hypothyroidism signs and symptoms include weight gain, decreased exercise capacity, constipation, fatigue, dry skin, hair loss, and bradycardia. Differential diagnosis should include depression, myalgic encephalomyelitis/chronic fatigue syndrome, and Addison disease, as these conditions share similar symptoms, such as fatigue, weight fluctuations, and difficulties with concentration, to hypothyroidism.^9^-^11^

## Hyperthyroidism in pregnancy

The incidence of hyperthyroidism during pregnancy is 0.2%, with overt hyperthyroidism defined as increased FT4 and low TSH and subclinical hyperthyroidism defined as asymptomatic low TSH and normal FT4.^2^,^12^ Graves disease is an autoimmune disorder with TSH receptor antibodies accounting for 95% of cases.^2^,^12^ Other less common causes of hyperthyroidism include gestational trophoblastic disease, nodular goiter, solitary toxic adenoma, viral thyroiditis, and pituitary or ovarian tumors.^2^ In pregnancy, hyperthyroidism has been associated with an increased risk of several complications, including spontaneous abortion, premature labor, low birth weight, stillbirth, preeclampsia, and maternal heart failure.^1^-^4^,^7^-^12^

Signs and symptoms of hyperthyroidism include failure to gain weight with adequate food intake, thyromegaly, exophthalmos, tachycardia, elevated resting pulse, nervousness, tremor, sweating, heat intolerance, proximal muscle weakness, increased frequency of bowel movements, decreased exercise tolerance, and hypertension.

Early elevations in hCG during pregnancy stimulate thyroid hormone output, which can lead to gestational transient thyrotoxicosis (GTT), also known as transient gestational hyperthyroidism. Graves disease and GTT have similar signs and symptoms to hyperthyroidism. Therefore, a thorough patient history, a physical exam, and indicated lab tests are imperative for an appropriate diagnosis.^13^ The differential diagnosis for hyperthyroidism may include TSH-secreting pituitary adenomas, trophoblastic tumors, hyperemesis gravidarum, multinodular goiter, familial nonautoimmune hyperthyroidism, autoimmune thyroiditis, and de Quervain thyroiditis.^4^

## Postpartum thyroiditis

The prevalence of postpartum thyroiditis varies greatly between 1.1% and 16.7%, with the condition defined as an abnormal TSH level in the first postpartum year in the absence of a toxic thyroid nodule or thyrotoxin receptor antibodies.^2^,^4^,^8^ Postpartum thyroiditis may occur after pregnancy loss (miscarriage, abortion, ectopic pregnancy) or after normal delivery. In postpartum thyroiditis, the clinical course may vary, with 25% of patients presenting with symptoms of hyperthyroidism followed by symptoms of hypothyroidism before then achieving an euthyroid state. Twenty to thirty percent of patients with postpartum thyroiditis go on to have persistent hypothyroidism.^14^ Patients with a history of thyroid disease, type 1 diabetes mellitus (T1DM), gestational diabetes, chronic hepatitis C, autoimmune disease, or a family history of thyroid disease are at increased risk of developing postpartum thyroiditis and should undergo TSH testing between 6 and 12 weeks postpartum.^2^,^14^ Patients who have postpartum depression, difficulties with milk production, or symptoms of thyroid disease, especially 3 to 6 months postpartum, should be tested for thyroid dysfunction.^1^,^2^,^14^ The signs and symptoms of hypothyroidism or hyperthyroidism are often dismissed as part of the normal transition from pregnancy to the postpartum period, which can lead to delays in diagnosis and treatment of postpartum thyroiditis.^13^-^15^

## Screening recommendations

Universal screening of asymptomatic pregnant patients for thyroid dysfunction is not currently recommended based on the available evidence.^2^,^15^ However, since as early as 2007, many experts have contended that targeted screening, which is currently recommended by most professional organizations, fails to identify many cases of thyroid disorders during pregnancy.^1^,^16^-^18^ The American College of Obstetricians and Gynecologists (ACOG) and ATA both cite the following as indications for targeted thyroid testing in pregnancy: family history of autoimmune thyroid disease; current thyroid therapy; goiter; and/or personal history of therapy for hyperthyroidism, autoimmune disease, T1DM, neck radiation, or postpartum thyroid dysfunction.^1^,^2^,^15^ The ATA also supports targeted thyroid testing for individuals who reside in iodine-deficient areas; have a body mass index (BMI) of 40 or higher, especially paired with other health conditions; are older than age 30 years; have decreased fertility; are using assisted reproductive technology; have had two or more previous pregnancies; have previously delivered an infant with thyroid disease; have previously delivered an infant preterm; and have experienced miscarriage.^19^ Preconception screening for thyroid dysfunction is recommended for high-risk patients, including those who are positive for thyroid peroxidase antibodies and patients planning for assisted reproduction.^2^

A recent survey of clinical practice patterns among endocrinologists, gynecologists, and obstetricians revealed that practice sometimes is not consistent with established professional guidelines, with 57% of endocrinologists and 71% of obstetricians reporting having conducted universal screening for thyroid disorders during pregnancy.^20^ A poster presentation at the 2023 ACOG annual meeting also posited that clinicians are not appropriately screening for thyroid disease regardless of the guidelines published by ATA and ACOG; this study, conducted at an Illinois hospital, showed that less than 50% of pregnant patients who met criteria for screening were appropriately screened for thyroid disease and that patients who were correctly screened per the ATA criteria had improved pregnancy outcomes.^18^ In some cases, clinical practices and individual clinicians may choose to screen patients differently than recommended in the guidelines based on their assessment of patient and population risk factors, signs, and symptoms.

## Evaluation of suspected thyroid disorders

Physical exam should include assessment of vital signs for tachycardia, bradycardia, and hypertension; assessment of weight and BMI for increase or decrease; observation of general appearance for diaphoresis, tremor, exophthalmos, and/or dry skin; and inspection and palpation of the thyroid gland for enlargement and/or nodules.

In the US, mild thyroid enlargement during pregnancy can be a typical finding; however, a significant goiter or distinct thyroid nodules are potentially abnormal and require assessment with thyroid function tests and thyroid ultrasonography. Although thyroid radionuclide scanning is contraindicated during pregnancy, a fine needle aspiration biopsy is safe if warranted.^2^

ACOG and ATA recommend a TSH level test as initial workup for thyroid disorders during pregnancy.^2^,^15^ In the presence of elevated TSH, ACOG and ATA next recommend assessment of FT4. In clinical practice, many labs now offer the ability to order a reflex FT4 automatically upon measure of an abnormal TSH, improving efficiency. If TSH is decreased, ACOG and ATA recommend assessment of both FT4 and total T3 (TT3). In the presence of risk factors and/or signs and symptoms, other tests such as free T3 (FT3), TT4, and thyroxine-binding globulin (TBG) may be warranted.^15^

Standard reference intervals for thyroid function parameters during pregnancy and postpartum vary greatly due to significant differences among populations studied, with geographic location, race, ethnicity, and socioeconomic status as contributing factors.^2^,^5^ The ATA recommends that population- and trimester-specific reference ranges for thyroid tests be used whenever possible. If this is not possible, the ATA advises that reference ranges from similar patient populations be used.^2^ Reference ranges also need to be assay-specific.^21^

TSH is usually lower during pregnancy, with a reduction of the lower limit of the reference range of about 0.4 mU/L and a reduction of the upper limit of about 0.5 mU/L in the first trimester relative to the typical nonpregnant range, which corresponds to about 0.1 to 4.0 mU/L.^2^,^3^ In the second and third trimesters, the TSH should increase, returning to, or close to, nonpregnant reference ranges. The ACOG guideline states that after the first trimester, nonpregnant reference ranges for TSH can be used, although the ATA guideline notes that levels remain lower in pregnant individuals than in nonpregnant individuals for the second and third trimesters.^2^,^15^ A study by La'ulu and Roberts found a second-trimester TSH reference interval of 0.15 to 3.11 mIU/L in a study of 3,064 US participants comprised of individuals who were White (42%), Hispanic (23%), Black (22%), and Asian (13%).^22^ A study by Dorizzi and colleagues found a second-trimester TSH reference interval of 0.68 to 4.07 mU/L in a study of 139 White women from northeast Italy.^21^

Before 16 weeks gestation, TT3, TT4, and TBG gradually increase compared with nonpregnant ranges. After 16 weeks gestation, TT3, TT4, and TBG are about 1.5 times higher than prepregnancy levels.^2^,^15^ Although a full discussion is outside of the scope of this article, it is important to note that FT4 test results for pregnant individuals are less reliable than they are for nonpregnant individuals. The ACOG guideline states that measurement of TT3 is preferred to FT3.^15^

Limited data are available for postpartum reference ranges in the literature, as many subjects are lost to follow-up; however, the literature states that most return to prepregnant levels. In the study by Dorizzi and colleagues, postpartum data were available for 55 of the participants, and a range of 0.56 to 3.42 mU/L was found.^21^

## Management

### Hypothyroidism

Management of hypothyroidism during pregnancy includes treating with incremental doses of levothyroxine that are adjusted based on the degree of TSH elevation. Serum TSH is measured every 4 weeks until midgestation and at least once around 30 weeks gestation; it is also measured at least every 4 to 6 weeks when medications are adjusted.^2^,^15^ Dosing recommendations for use of levothyroxine in overt hypothyroidism in pregnancy can be found in the ATA and ACOG guidelines.^2^,^15^ If taking levothyroxine for pregestational hypothyroidism, the dose should be increased by about 20% to 30% upon confirmation of pregnancy.^2^ The treatment goal for hypothyroidism in pregnancy is to achieve a TSH level between the lower reference limit and 2.5 mU/L. Antenatal surveillance with fetal nonstress test and biophysical profile is not recommended in pregnant patients with hypothyroidism that is well controlled with medication, but it may be considered in patients with coexisting maternal or obstetric indications.

Levothyroxine is indicated in some individuals with subclinical hypothyroidism in pregnancy; providers should refer to the ATA guideline for more information.^2^

After delivery, the levothyroxine dose should be decreased to the prepregnancy dose for patients with pregestational hypothyroidism, and a TSH level should be checked at 6 weeks postpartum. In many, but not all, patients diagnosed with hypothyroidism during pregnancy, thyroid function returns to normal in the postpartum period, and levothyroxine can be discontinued. If it is discontinued, a TSH level should be checked about 6 weeks later.^2^ A vast amount of variability in thyroid levels is possible during the postpartum period, especially in patients who are breastfeeding, as prolactin—the most important hormone in lactation—is regulated by the pituitary gland and affected by TSH levels. Exclusivity and duration of breastfeeding are other factors that may also contribute to the variability of thyroid levels in the postpartum period. A recent study by Li and colleagues reported that among patients diagnosed with subclinical hypothyroidism during pregnancy, 38.9% went on to have long-term hypothyroidism, including some who had normal thyroid function at 6 weeks postpartum.^23^

### Hyperthyroidism

Management of hyperthyroidism includes treating with incremental doses of propylthiouracil in the first trimester, changing to methimazole in the second trimester. Propylthiouracil should be discontinued in the second trimester due to risk of maternal liver damage, whereas methimazole should not be used in the first trimester due to risk of birth defects during that time.^2^,^15^ Dosing for both drugs is based on TSH, FT4, and TT3 levels, which should be measured every 2 weeks until stable, with a treatment goal of FT4 in the upper third of the normal range.^4^,^15^ Once stabilized in the high normal range, thyroid labs should be checked every 4 weeks.^15^ Antenatal testing, such as fetal ultrasonography, should be performed in patients with poorly controlled hyperthyroidism, typically in conjunction with maternal-fetal medicine, to assess for signs of fetal hyperthyroidism, such as fetal growth restriction, hydrops, goiter, and cardiac dysfunction.^4^,^15^

Treatment of subclinical hyperthyroidism in pregnancy is generally not recommended.^2^,^15^

### Postpartum thyroiditis

Providers may consider obtaining TSH levels at 6 to 12 weeks and 3 to 6 months postpartum for patients at high risk for postpartum thyroiditis, such as those with T1DM or previous postpartum thyroiditis.^19^ During management of the hyperthyroid phase of postpartum thyroiditis—which is typically caused by autoimmune destruction of the thyroid resulting in release of stored thyroid hormone—antithyroid medications are not beneficial. However, beta-blockers may be used if the patient is symptomatic.^2^,^4^,^15^ It is important to differentiate the hyperthyroid phase of postpartum thyroiditis from Graves disease, since Graves disease requires antithyroid therapy. Differentiation is possible with referral for radioactive iodine uptake scan. However, this scan is contraindicated during breastfeeding, and close contact with others must be limited after its performance due to the scan's radioactivity.^2^,^15^ In the hypothyroid phase of postpartum thyroiditis, patients may be treated with levothyroxine if symptomatic.^2^,^15^,^19^ In most cases of postpartum thyroiditis, thyroid function levels return to normal within 12 to 18 months. However, a small percentage of patients may not recover, leading to permanent hypothyroidism requiring lifelong supplementation.^2^,^15^,^19^

## Patient education

### Hypothyroidism

Patients should be educated on the symptoms of hypothyroidism including lack of energy and feeling fatigued easily; feeling cold easily; developing coarse or thin hair; and constipation. If not treated, hypothyroidism can weaken and slow the heart, often causing patients to feel out of breath or tired when exercising and causing edema in the ankles.^4^ Untreated hypothyroidism can also increase BP and raise cholesterol, both of which increase the risk of heart disease. Patients who are prescribed thyroid hormone medication should be educated on the importance of taking it every day. After taking the medication for 4 to 6 weeks, follow-up blood tests are needed to ensure appropriate levels. The medication dose may require adjustment depending on test results. Levothyroxine is the medication of choice; patients should be advised to take this medication by itself on an empty stomach, ideally 1 hour before breakfast daily as well as 4 to 5 hours before or after taking any drugs known to interfere with its absorption, such as certain medications, certain vitamins, calcium, and iron.^24^ Most people with permanent hypothyroidism need to be on thyroid medication for the rest of their life, and patients should be counseled accordingly.

### Hyperthyroidism

Patients should be educated on the common symptoms of hyperthyroidism including feeling tired or weak; losing weight, even when eating normally; having a fast or uneven heartbeat; sweating excessively and having trouble dealing with hot weather; feeling worried; and trembling. There may be a noticeable enlargement of the thyroid gland or palpable nodules on the thyroid. Patients of reproductive potential who have hyperthyroidism should also receive preconception counseling: If hyperthyroidism is present prepregnancy, a euthyroid state should be achieved before trying to conceive to decrease risk to the fetus.^13^

### Postpartum thyroiditis

Thyroiditis after pregnancy can cause symptoms of hyperthyroidism followed by hypothyroidism; isolated symptoms of hyperthyroidism; or isolated symptoms of hypothyroidism.^2^,^4^ Patients with the condition should be educated about the expected clinical course, signs and symptoms, and recommended testing and treatments.

### Iodine intake

Patient education should include information on iodine intake. The ATA recommends that all pregnant and breastfeeding women consume 250 mcg of iodine daily, including a daily oral supplement containing 150 mcg of iodine (in the form of potassium iodine).^2^ Women planning pregnancy should also consume a daily oral supplement with 150 mcg of iodine. Sufficient iodine intake is also important while breastfeeding to ensure that infants receive iodine in breast milk. Good dietary sources of iodine include dairy, seafood, eggs, meat, poultry, and iodized salt.^6^,^24^ Caution patients that too much iodine from diet and dietary supplements such as seaweed during pregnancy or breastfeeding may be harmful and can lead to fetal or infant hypothyroidism and goiter.^2^,^24^

## Consultation and referral

For the advanced practice provider, consultation with an endocrinology specialist is recommended upon the diagnosis of hypothyroidism, hyperthyroidism, postpartum thyroiditis, or goiter in pregnancy. Referral should be made to the appropriate specialist in the event of poorly controlled thyroid function test levels and/or worsening of symptoms. During pregnancy, referral to and/or comanagement with maternal-fetal medicine as indicated—but especially for patients with elevated TSH receptor antibodies, uncontrolled hyperthyroidism, or a history of irradiation for hyperthyroidism on replacement therapy—are key to improving maternal and fetal outcomes.

## Implications and conclusion

As noted, variations in reference intervals for thyroid function parameters are common throughout the literature because of differences in methods used and populations included in these studies.^21^ Defining standard population-based trimester-specific reference intervals for thyroid function parameters may be helpful from both academic and clinical points of view and may suggest directions for further research. Although current guidelines recommend the assessment of thyroid function parameters in pregnancy using trimester- and instrumentation reagents-specific reference intervals, such recommendations are rarely implemented in practice, as clinicians usually accept the intervals suggested by the clinical lab manufacturers.

It is also noteworthy that iodine intake has been inadequately evaluated throughout the literature. Urinary iodine concentration to assess the iodine status of the study population has not been measured in many studies, and when it has been measured, it has not been assayed with standard reference technology.^21^ Future research should be undertaken to explore this topic.
